# Phosphorylation regulation of cardiac proteins in *Babesia microti* infected mice in an effort to restore heart function

**DOI:** 10.1186/s13071-022-05233-7

**Published:** 2022-03-21

**Authors:** Xiaohong Yang, Ningmei Wang, Shuguang Ren, Yuhong Hu, Han Wang, Aimeng Ji, Lihui Cao, Mengxue Li, Jingze Liu, Hui Wang

**Affiliations:** 1 Ministry of Education Key Laboratory of Molecular and Cellular Biology, Hebei Key Laboratory of Animal Physiology, Biochemistry and Molecular Biology, College of Life Sciences, Hebei Normal University, Shijiazhuang, Hebei China; 2 Department of Pathogenic Biology, College of Basic Medicine, Hebei Medical University, Shijiazhuang, Hebei China; 3 The Fourth Hospital of Hebei Medical University, Shijiazhuang, Hebei China; 4 Instrumental Analysis Center, Hebei Normal University, Shijiazhuang, Hebei China; 5 Animal Disease Control Center of Zhengding County, Shijiazhuang, Hebei China

**Keywords:** Apoptosis, *Babesia*, Heart injury, Oxidative phosphorylation, Proteomics

## Abstract

**Background:**

*Babesia* is a common protozoan parasite that infects red blood cells. In mice infected with *Babesia microti*, the red blood cells were lysed, resulting in decreased oxygen-carrying capacity. To compensate for low blood oxygen levels, stress on the heart was greatly increased. Babesiosis induces a variety of pathologies; meanwhile, heart tissues initiate self-repair responses to babesiosis-induced tissue damage to restore heart function.

**Methods:**

To discover the molecular mechanisms of the damage and self-repair in the heart after *B. microti* infection in mice, we investigated the changes in protein expression and phosphorylation modification levels in heart tissues at 0, 5, 8, 11, and 19 days post-infection using data-independent acquisition (DIA) quantitative proteomics.

**Results:**

The numbers of global proteins we identified were 1934, 1966, 1984, 1989, and 1955 and of phosphopeptides were 5118, 5133, 5130, 5133, and 5140 at 0, 5, 8, 11, and 19 days, respectively, in heart cells after infection with *B. microti*. The results showed that after *B. microti* infection the differentially expressed proteins in mice mainly include fibrinogen α (Fgα), fibrinogen β (Fgβ), Serpina1b, Serpina1c, cathepsin Z, cytochrome c oxidases (COXs), RPS11, and RPS20. The proteins with phosphorylation changes mainly include 20-kDa light chain of myosin II (MLC20), myosin light chain kinase (MLCK), mitogen-activated protein kinase 14 (MAPK14), and Akt1. These proteins were mainly involved in coagulation processes, cell apoptosis, oxidative phosphorylation, and ribosomes.

**Conclusions:**

The coagulation cascade-related proteins, apoptosis-related proteins, oxidative phosphorylation-related proteins, and other types of proteins are all involved in the damage and self-repair process in the heart after *B. microti* infection. These results offer a wealth of new targets for further exploration into the causes of heart disease induced by *Babesia* infection and are of great significance for novel drug development and new opportunities for targeted therapies.

**Graphical Abstract:**

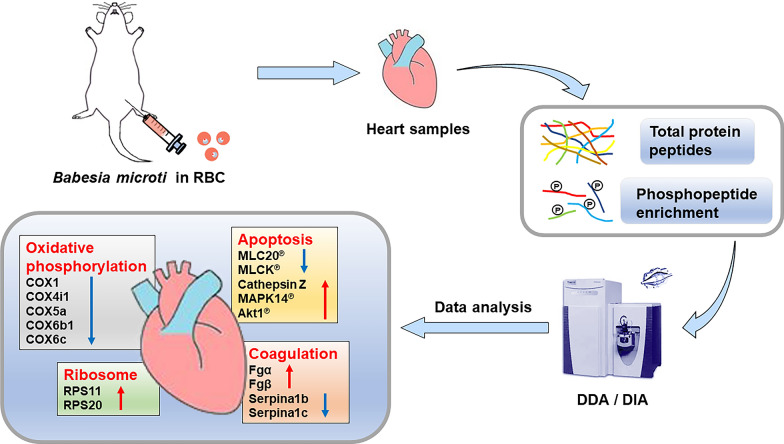

**Supplementary Information:**

The online version contains supplementary material available at 10.1186/s13071-022-05233-7.

## Background

Babesiosis is a zoonotic disease caused by the protozoan *Babesia*, which infects the red blood cells (RBCs) of mammals, birds, and occasionally humans [1, 2]; the main *Babesia* species identified to date that cause babesiosis in humans include *Babesia microti*, *Babesia divergens*, *Babesia duncani*, and *Babesia venatorum* [3]. *Babesia* can be transmitted to humans and other mammals through the blood-sucking activity of vector ticks as well as through placenta or blood transfusion [3]. Due to the widespread distribution of ticks and the difficulty of treatment, babesiosis is widely transmitted among various wild and domestic animals [1]. Furthermore, the number of confirmed cases of human babesiosis worldwide is increasing annually [5].

*Babesia* infection in humans can cause haemolytic anaemia and develop clinical symptoms, such as fever, sweating, and muscle pain; multiple organ diseases, such as hepatosplenomegaly [6], acute nephritis [7], and myocarditis [8]; and acute respiratory distress syndrome, disseminated intravascular coagulation, heart failure, and even death in severe cases depending on the immunocompetence of the infected individual [9–11]. Infection with *Babesia* can quickly activate the immune defense system to fight it, and some infected patients can recover without specific therapeutic intervention [12]. However, unfortunately, the underlying mechanisms of autoimmune regulation that allow hosts to heal themselves remain unclear.

Elucidating the immunoregulatory mechanisms of hosts can provide an important theoretical basis for the treatment and prevention of parasitic diseases. In the past decade, mass spectrometry has been widely used as a powerful biological research technology in early disease diagnosis, pathology research, treatment, and drug target screening [13]. With the continuous innovation of proteomic technology, more accurate and high-throughput protein quantitative studies are available [14]. It is worth mentioning that many studies on protein post-translational modification, which were difficult to carry out before, have also been well solved [15–17]. Protein phosphorylation is an important post-translational modification in organisms; it is involved in regulating all life activities, including cell proliferation, signal transduction, metabolism, disease occurrence, and so on [18]. With the rapid development of mass spectrometry technology, the invented high-energy collision dissociation (HCD) technology solves the disadvantage of low-energy fragmentation of phosphopeptides in previous mass spectrometry, making phosphopeptides easier to identify [19, 20]. This has accelerated the research on phosphorylated proteins in recent years [21–23]. In the present study, to investigate the molecular mechanisms involved in fatal cardiac injury and myocardial repair caused by *B. microti* infection, the changes of total proteins and phosphorylation modification of these proteins were first analysed using a data-independent acquisition (DIA) quantitative proteomic approach. Our findings lay the foundation for the treatment of heart damage caused by *Babesia* infection and future studies of the mechanisms of immune regulation and self-repair of the heart.

## Methods

### Mouse model of *Babesia* infection and dissection of mice tissues

*Babesia microti* (ATCC PRA-99™) was generously provided by the Institute of Laboratory Animal Science, Chinese Academy of Medical Sciences. Red blood cells (1.8 × 10^7^ preserved in liquid nitrogen) of mice containing *B. microti* were intraperitoneally injected into 6-week-old female BALB/c mice [24]. For quantitative assessments of total protein, there were five groups—5, 8, 11, and 19 days after infection and control group (injected with physiological saline). Four biological replicates were included for each group, and a total of 20 mice were used. The same numbers of mice and grouping methods were used for quantitative assessments of phosphorylated protein. Blood was collected from the tail tip every day. Parasitaemia was monitored by examination of Giemsa-stained blood smears and detection of *B. microti* DNA by PCR [25]. After 5, 8, 11, and 19 days of continuous feeding, mice were killed by CO_2_ inhalation followed by cervical dislocation. Heart tissues were promptly dissected and rapidly rinsed in deionized water to remove excess blood, then quickly frozen, and stored at − 80 °C for subsequent experiments. All methods were approved by the Animal Ethics Committee of Hebei Normal University (no. 165031). The procedures of animal feeding and material collection were completed in the second level Biosafety Laboratory of the fourth hospital of Hebei Medical University [26].

### Protein extraction and proteomic sample preparation

**Fig. 1:**
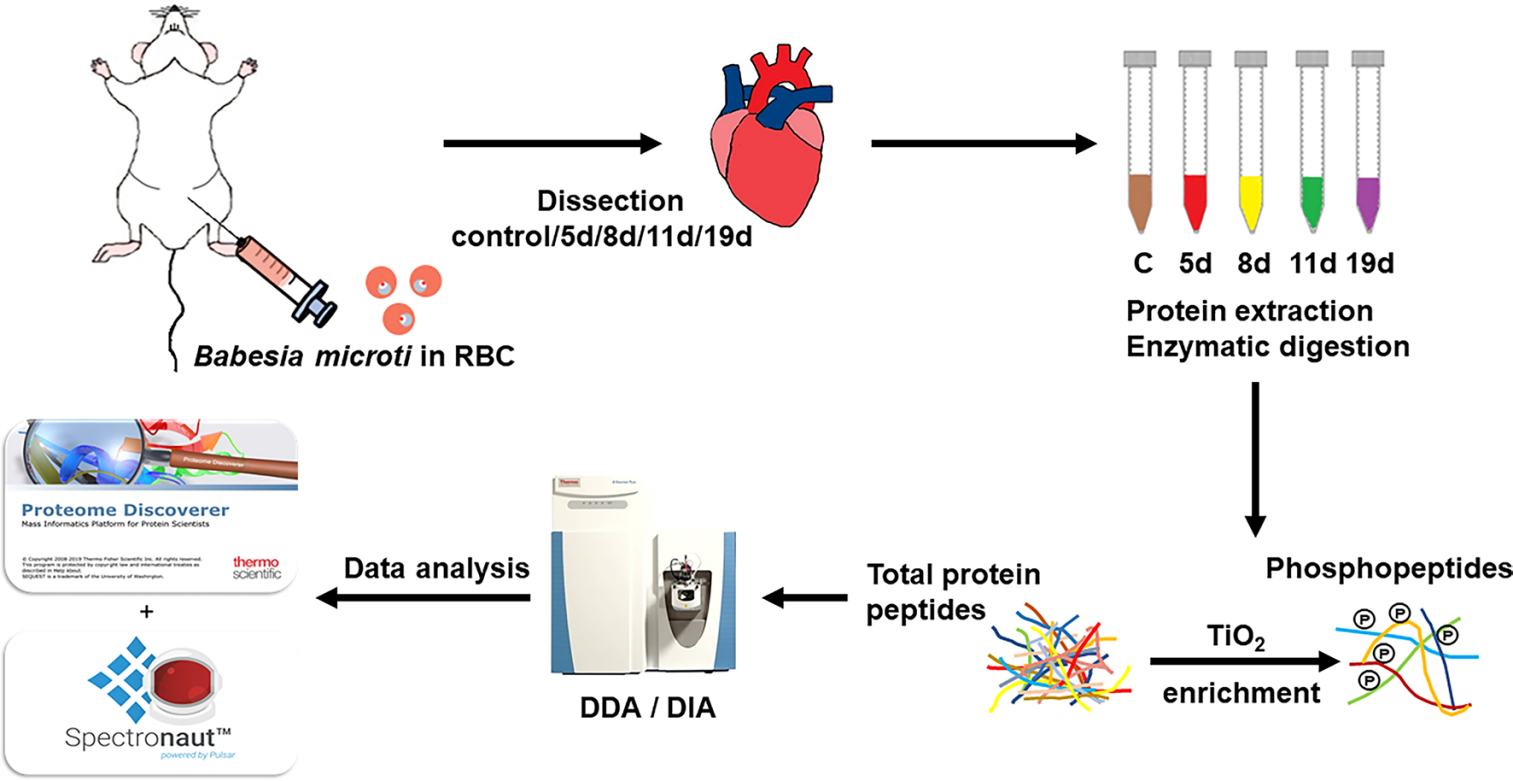
Overall experimental design for proteomic analysis of total proteins and phosphorylated proteins in heart tissues of mice infected with *B. microti*

Heart tissues from different stages were ground in PBS (0.1 M, pH 6.8) containing a mixture of PhosSTOP and cOmplete EASYpack protease inhibitor cocktail (Roche, Germany). Then, the homogenate was centrifuged for 15 min (4 °C, 12,000*g*), and the supernatant was transferred to a new centrifuge tube. The same volume of Tris-saturated phenol (pH 7.8) was added, and the mixture was vortexed and centrifuged for 20 min. Later, the same volume of 50 mM Tris–HCl (pH 8.0) was added, and the mixture was centrifuged. After removing the upper aqueous phase, the protein content was precipitated by adding a certain volume of 0.1 M ammonium acetate in methanol and stored at − 20 °C overnight. After centrifugation, the supernatant was discarded. The protein pellet at the bottom of the tube was washed with methanol twice, and the extracted proteins were lyophilized. Then, 10 mM dithiothreitol (Promega, USA), 20 mM iodoacetamide (Promega, USA), and trypsin (1:20 w/w, Promega, USA) were added to the extracted protein globules successively. The treated proteins were incubated at 37 °C overnight to be digested into peptides and then desalted with C18 solid-phase extraction column (CNW®, China), according to the manufacturer’s instructions. BCA protein assay kit (Pierce Biotechnology) was used to confirm the peptide concentration. The digestion efficiencies of protein were determined by Q Exactive HF (Thermo Fisher, USA) [27]. The whole experimental flow of quantitative proteomics sample processing and identification is shown in Fig. 1.

### Enrichment of phosphorylated peptides by TiO_2_

Aliquots of TiO_2_ (GL Sciences, Japan) beads were washed three times with a 50% acetonitrile (ACN) buffer containing 2% trifluoroacetic acid (TFA) saturated with glutamic acid. TiO_2_ beads and desalinated peptides were mixed and gently shaken in the same buffer for 1 h at room temperature. Then, the beads were washed with 50% ACN and 50% ACN containing 20 mM ammonium acetate. The phosphopeptides were eluted with 200 μl 0.3 M NH_4_OH once and eluted with 200 μl 0.5 M NH_4_OH twice from the beads. Four biological replicates were performed in this experiment.

### RP-HPLC for peptides classification separation

The digested peptide samples from the control group and each treatment group (5, 8, 11, and 19 days) were mixed in equal proportions. Then, the mixed peptides were separated by high-pH reversed-phase high-performance liquid chromatography (RP-HPLC) (Waters e2695, USA). The Durashell C18 column (5-μm particle size, 100-Å aperture, 4.6 mm × 250 mm, Agela, China) was used to separate peptides with a flow rate of 1 ml/min. The changing mode of mobile phase ratio was as follows: 2% solvent B (100% ACN contains 5 mM ammonium formate, pH 10.0) and 98% solvent A (100% water contains 5 mM ammonium formate, pH 10.0) for 10 min and then from 2 to 50% solvent B within 70 min. During this time, peptides eluted per minute were collected separately. In total, 60 fractions were collected. The 60 fractions were mixed into ten tubes according to a certain order (1, 11, 21, 31, 41, 51; 2, 12, 22, 32, 42, 52, ……). Then, the samples were lyophilized and stored at – 80 °C.

### DDA spectral library construction

Each sample was resuspended in solvent A (99.9% H_2_O, 0.1% formic acid), and the appropriate proportion of iRT (Spectronaut, Switzerland) was added. UPLC M-Class system (Waters, USA) was used for peptide separation before spraying into the mass spectrometry. The separation was carried out in a dual column mode, with the front C18 RP trap column (5-μm particle size, 100-Å pore size, 180-μm ID × 20-mm length; Waters, USA) and the back C18 RP analytical column (1.8-μm particle size, 100-μm ID × 150-mm length; Waters, USA). The flow rate of the whole elution process was set as 300 nl/min using a linear gradient. The elution procedure was as follows: start with 2–8% solvent B in 6 min and then 8–35% solvent B in next 114 min (solvent A: 99.9% H_2_O, 0.1% formic acid; solvent B: 99.9% ACN, 0.1% formic acid). Q Exactive HF (Thermo Fisher, USA) mass spectrometer was used for DDA identification to construct spectral library, and machine operating parameters were set as previously described [28]. The construction of DDA spectral library and identification method of mass spectrometer for phosphopeptides were the same as described above for the identification method of total proteins. Finally, the results of ten raw files identified were combined and searched by Proteome Discoverer (version 2.2, Thermo Fisher, USA) software. Data search parameters were set as previously described [28]. The database was derived from *Mus musculus* protein sequences from UniProt (2017/12/07, 16,944 sequences), and trypsin, human keratins, and *Babesia* protein sequences were set as contaminated database.

### DIA quantitative proteomic analysis

DIA method was used to quantify the samples of control group and different infection stages. The chromatographic conditions for DIA were the same as those for DDA spectral library construction. Mass spectrometry parameters were set as follows: (i) DIA mode; scanning range set to 350–1200 m/z; resolution of the precursor ion set to 60,000; automatic gain control (AGC) target set to 3 × 10^6^; and maximum ion injection time (maximum IT) set to 50 ms; (ii) HCD normalised collision energy set to 27%; (iii) MS2 scanning set to 34 consecutive windows (26 m/z) and 1 m/z overlap between 2 adjacent windows; (iv) MS2 scan resolution set to 30,000; AGC target set to 1 × 10^6^; and maximum IT set to auto. Spectronaut software (version 11.0, Switzerland) was used to calculate the DIA result, which used the default parameters. FDR was set to 1%.

### Bioinformatics analysis

The differentially expressed proteins and phosphopeptides were analysed using bioinformatics methods. We defined changes in expression level based on the following ratios of 5 days/0 days (control), 8 days/0 days, 11 days/0 days, and 19 days/0 days. The protein expression level and phosphorylated peptides were considered upregulated when the ratio was > 1.5 and downregulated when the ratio was < 0.67 (log2 > 0.58 or log2 < – 0.58) [13]. GProX was used to cluster the proteins with similar expression change characteristics [29]. Principal component analysis (PCA) was carried out by Online analysis software (https://www.omicsolution.org/wkomics/main/). PANTHER software was used in the functional category of Gene Ontology (GO) (http://pantherdb.org/). Pathways associated with differential protein expression were identified by Kyoto Encyclopedia of Genes and Genomes (KEGG) database (http://www.kegg.jp/kegg/).

## Results

### Quantitative results of total protein in the heart of mice before and after *B. microti* infection

**Fig. 2:**
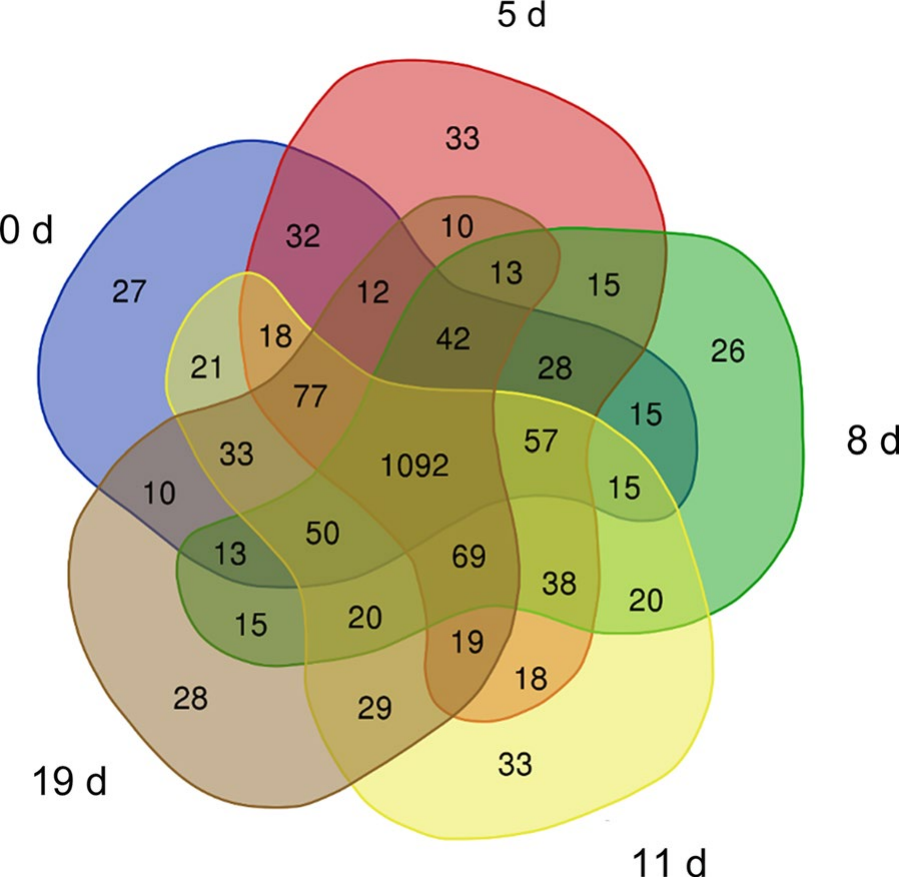
Venn diagram analysis of the total proteins identified at different stages. Blue: control group (0 days). Red: group infected for 5 days. Green: group infected for 8 days. Yellow: group infected for 11 days. Grey: group infected for 19 days

**Fig. 3:**
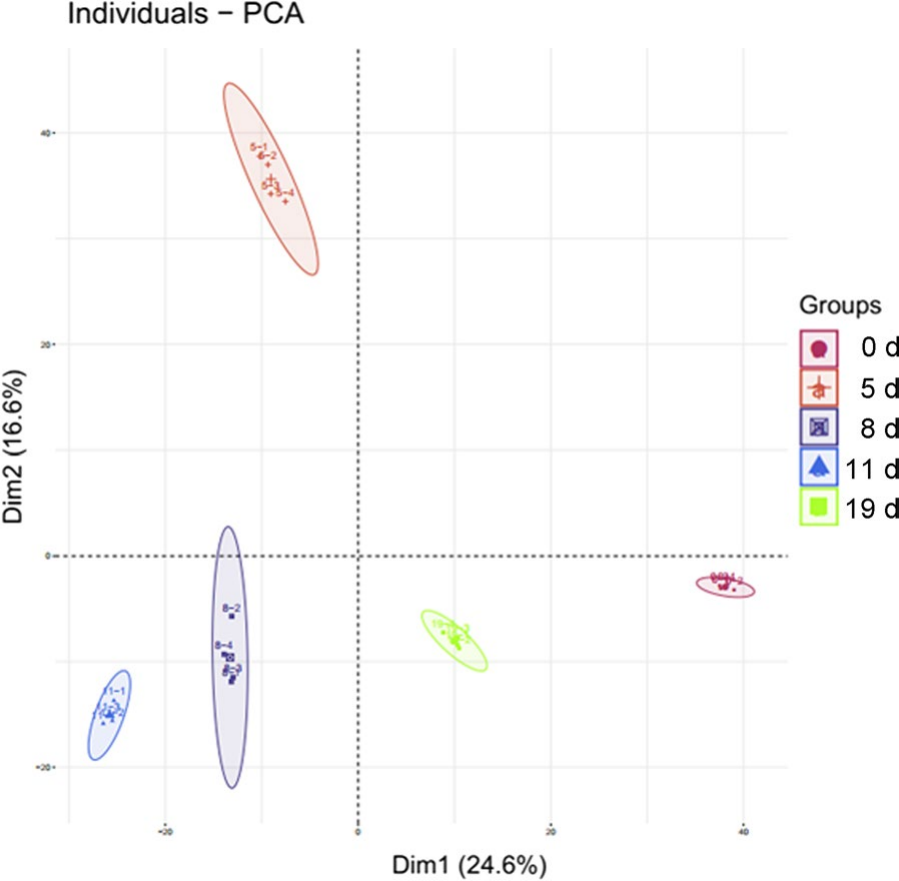
PCA diagram of four repeated proteomics data identified at different stages

The numbers of proteins identified at 0, 5, 8, 11, and 19 days after infection with *B. microti* were 1934, 1966, 1984, 1989, and 1955, while 1542, 1573, 1528, 1609, and 1532 proteins had a coefficient of variation (CV) < 20%, respectively (Additional file 7: Table S1). Venn analysis was performed on identified proteins at the five stages, and 1092 proteins were identified in all five stages (Fig. 2). Principal component analysis (PCA) was performed on the four replicates of the five groups of data (Fig. 3). The results showed that experimental data had significant differences between all groups, whereas the differences of protein identification among four biological replicates at different stages post-infection were small, indicating high reproducibility of data in each group. The mass spectrometry proteomics data have been deposited in the ProteomeXchange Consortium (http://proteomecentral.proteomexchange.org) via the iProX partner repository [30] with the dataset identifier PXD029452. ‍

### Cluster analysis of differentially expressed total proteins

**Fig. 4:**
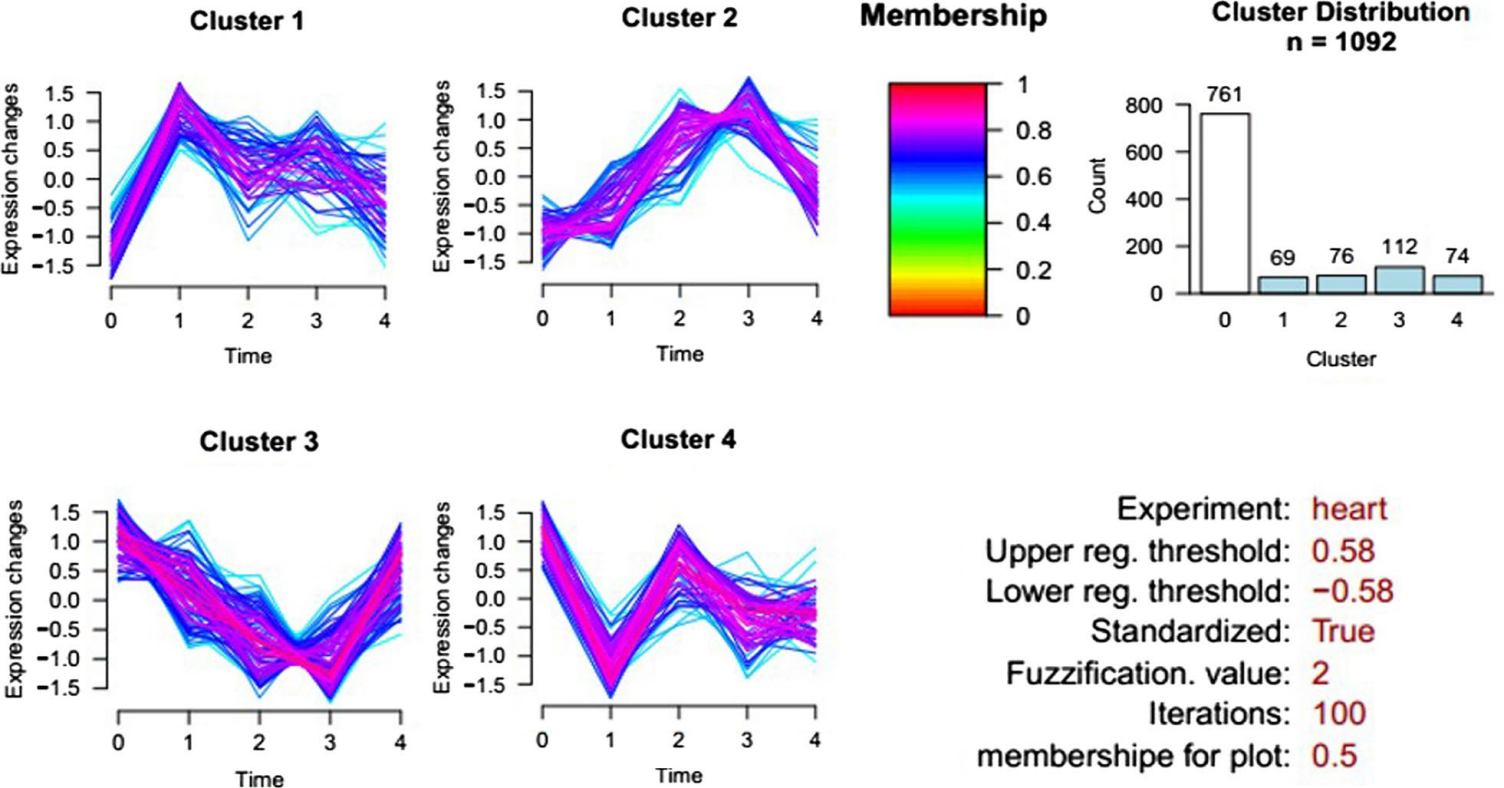
Cluster analysis of interaction proteins

Cluster analysis software was used to intuitively analyse the variation trend of 1092 intersection proteins identified over five stages (Fig. 4). The ratio results of 5 days/0 days, 8 days/0 days, 11 days/0 days, and 19 days/0 days (Additional file 8: Table S2) were used to carry out cluster calculation and analysis. The analysis results showed that 331 proteins' expression levels changed in different periods after infection (Additional file 9: Table S3). We divided the variation trend of these differentially expressed proteins into four types: The expression level of 69 proteins in Cluster 1 was upregulated at 5 days after *B. microti* infection and then slightly downregulated. Seventy-six proteins in Cluster 2 were upregulated at 5, 8, and 11 days after infection and downregulated at 19 days. Cluster 3 had the most proteins; 112 proteins were downregulated at 5, 8, and 11 days after *B. microti* infection and upregulated at 19 days. Seventy-four proteins in Cluster 4 were downregulated at 5 days and quickly upregulated at 8 days and then slightly downregulated.

### GO annotation analysis of differentially expressed proteins in four clusters

**Fig. 5:**
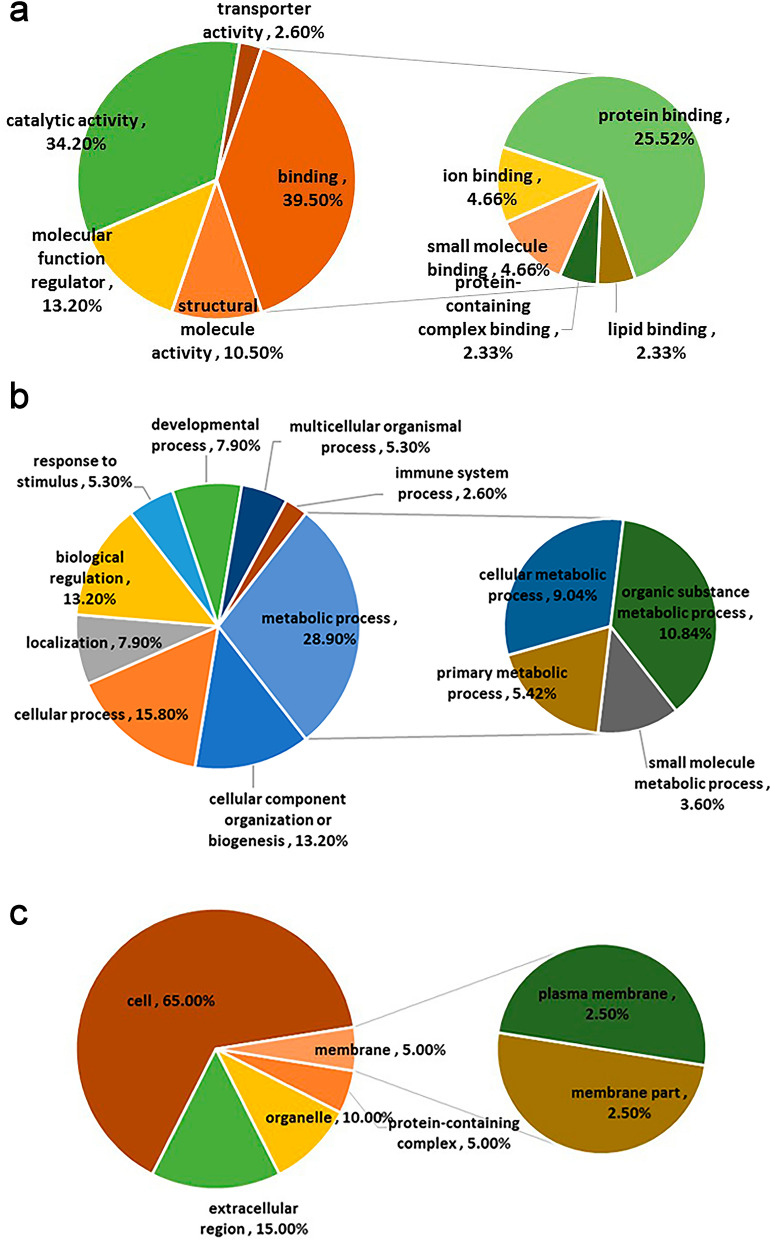
GO analysis of differential expressed proteins in Cluster 1. **a** Molecular function; **b** biological process; **c** cell component

The differentially expressed proteins in the four clusters obtained by cluster analysis were analysed by GO annotation. These proteins were classified into three categories: molecular function, biological process, and cell component (Fig. 5 and Additional file 1: Fig. S1, Additional file 2: Fig. S2, Additional file 3: Fig. S3). In Cluster 1, in terms of protein molecular function, 34.2% of Cluster 1 had catalytic activity and 39.5% had binding activity, of which protein binding function accounted for the largest proportion, 25.52%. Of the total proteins in Cluster 1, 28.9% of the proteins were involved in metabolic processes, including organic metabolism and cell metabolism, 13.2% in cell regulation, and 15.8% in cell biological processes. Of these proteins, 65% were located in the cytoplasm, and 15% were located outside the cell. There were 2.5% proteins on the plasma membrane and other membrane structures.

### KEGG pathway analysis of differentially expressed proteins

**Fig. 6:**
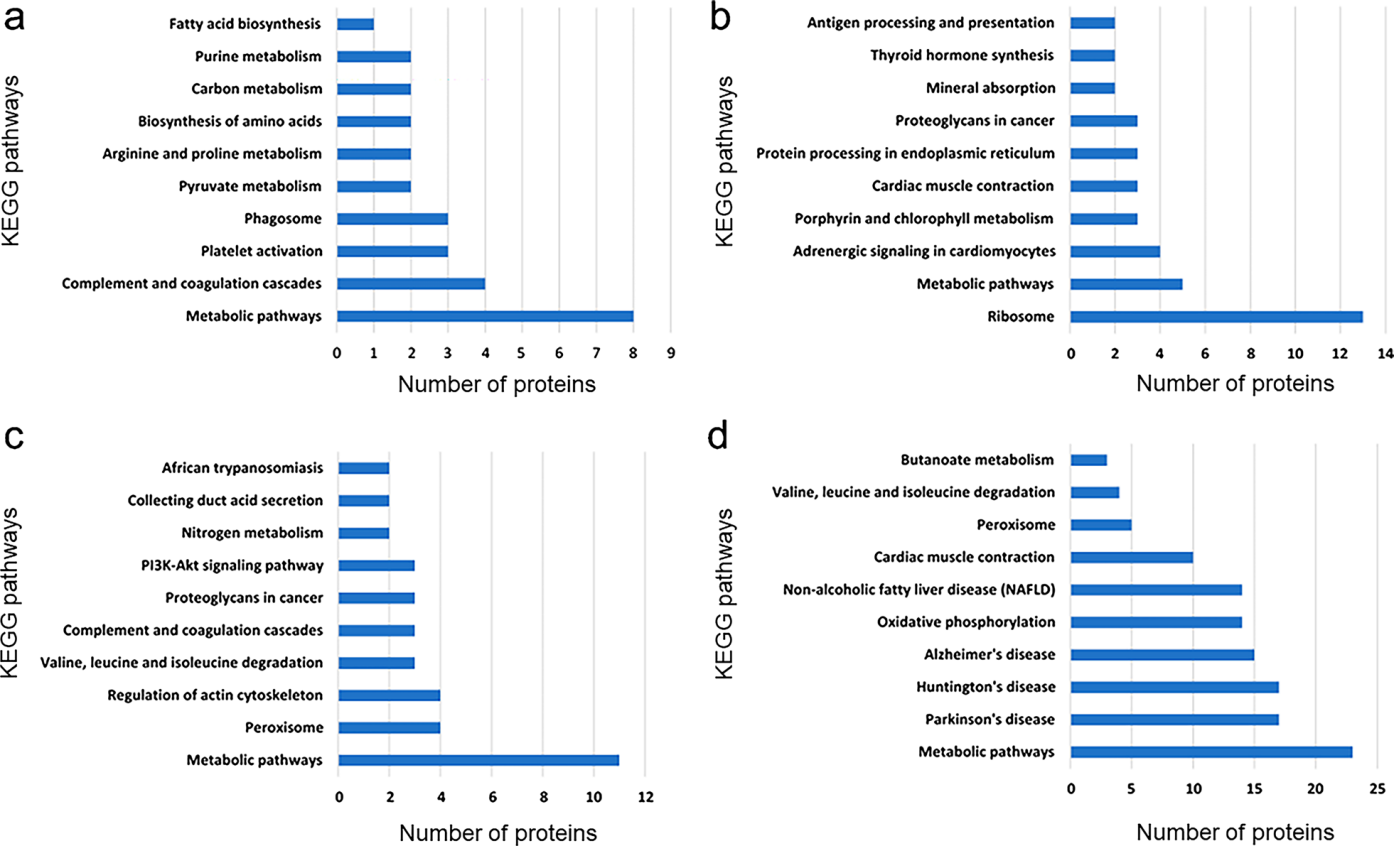
KEGG pathway analysis and quantitative statistics for differentially expressed proteins in each cluster. **a** KEGG enrichment results of proteins in Cluster 1; **b** KEGG enrichment results of proteins in Cluster 2; **c** KEGG enrichment results of proteins in Cluster 3; **d** KEGG enrichment results of proteins in Cluster 4

The differential proteins in each cluster were enriched by KEGG and the number of pathways was counted (Fig. 6). In each group, there were many proteins identified with predicted involvement in the metabolic process. For example, four proteins in Cluster 1 were involved in the process of complement and coagulation, and three proteins were involved in platelet activation. These proteins may be related to coagulation. In addition, two proteins were involved in pyruvate metabolism, two proteins were involved in arginine proline metabolism, and two proteins were involved in amino acid synthesis. These were related to amino acid metabolism and may be related to protein synthesis. There were also three species involved in autophagy, which may be related to cell damage.

### Quantification of phosphorylated proteins

The phosphopeptides in each group identified by mass spectrometry were analysed by Spectronaut™ 11 software. Due to the TiO_2_ enrichment process, the quantitative results of the proteins identified with phosphopeptides were not authenticated, rather focus was placed on the different mass spectrum intensities of phosphopeptides to determine the change of phosphorylation modification degree of phosphorylated proteins. The numbers of phosphopeptides identified on days 0, 5, 8, 11, and 19 after *B. microti* infection were 5118, 5133, 5130, 5133, and 5140, respectively (Additional file 10: Table S4).

**Fig. 7:**
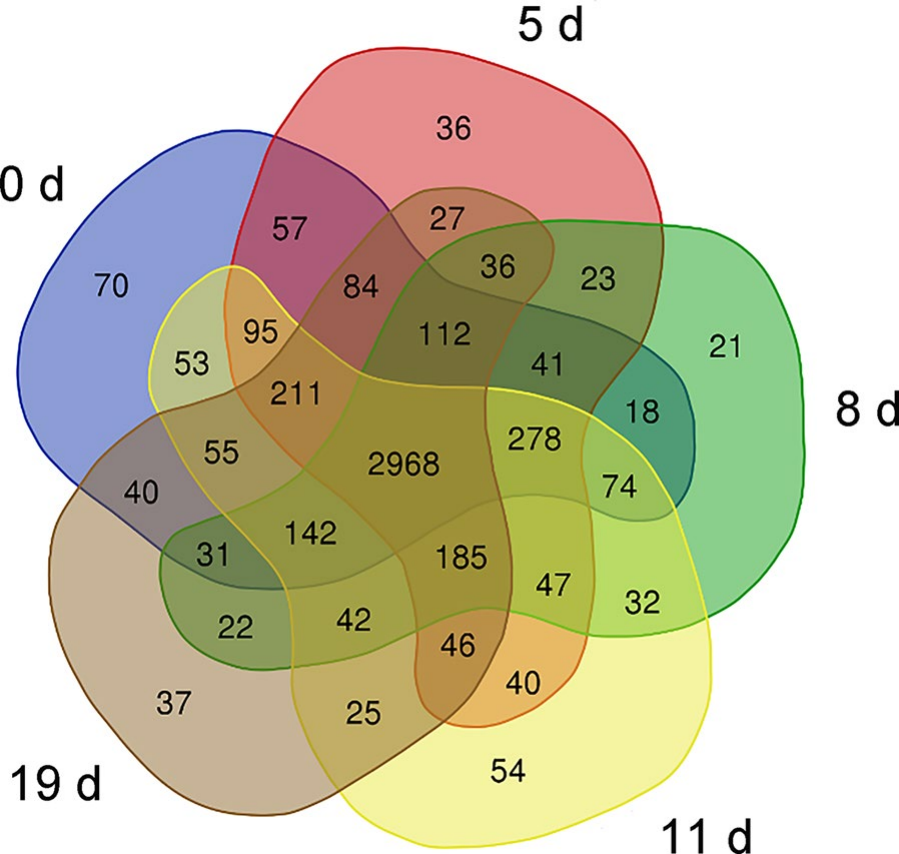
Venn diagram analysis of the phosphopeptides identified at different stages. Blue: control group (0 d). Red: group infected for 5 days. Green: group infected for 8 days. Yellow: group infected for 11 days. Grey: group infected for 19 days

Venn analysis was performed on the phosphopeptides identified in each stage (Fig. 7). The intersection of phosphopeptides identified in the five stages was 2968 (Additional file 11: Table S5), corresponding to 1751 phosphorylated proteins.

**Fig. 8:**
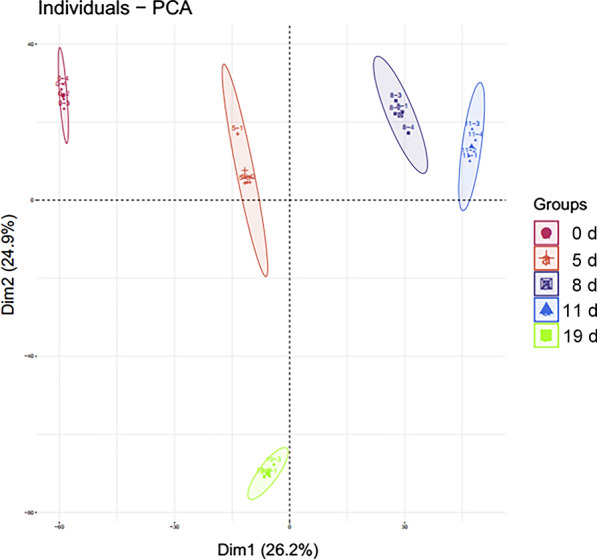
PCA diagram of four repeated phosphopeptides identified at different stages

Principal component analysis (PCA) of the proteins identified in the five stages (four biological replicates in each stage) (Fig. 8) reveals little difference in protein identification among the four biological replicates in each stage, indicating that the reproducibility of each group of data was high; as expected, there were differences in proteins between the different stages.

### Cluster analysis of differentially expressed phosphopeptides

**Fig. 9:**
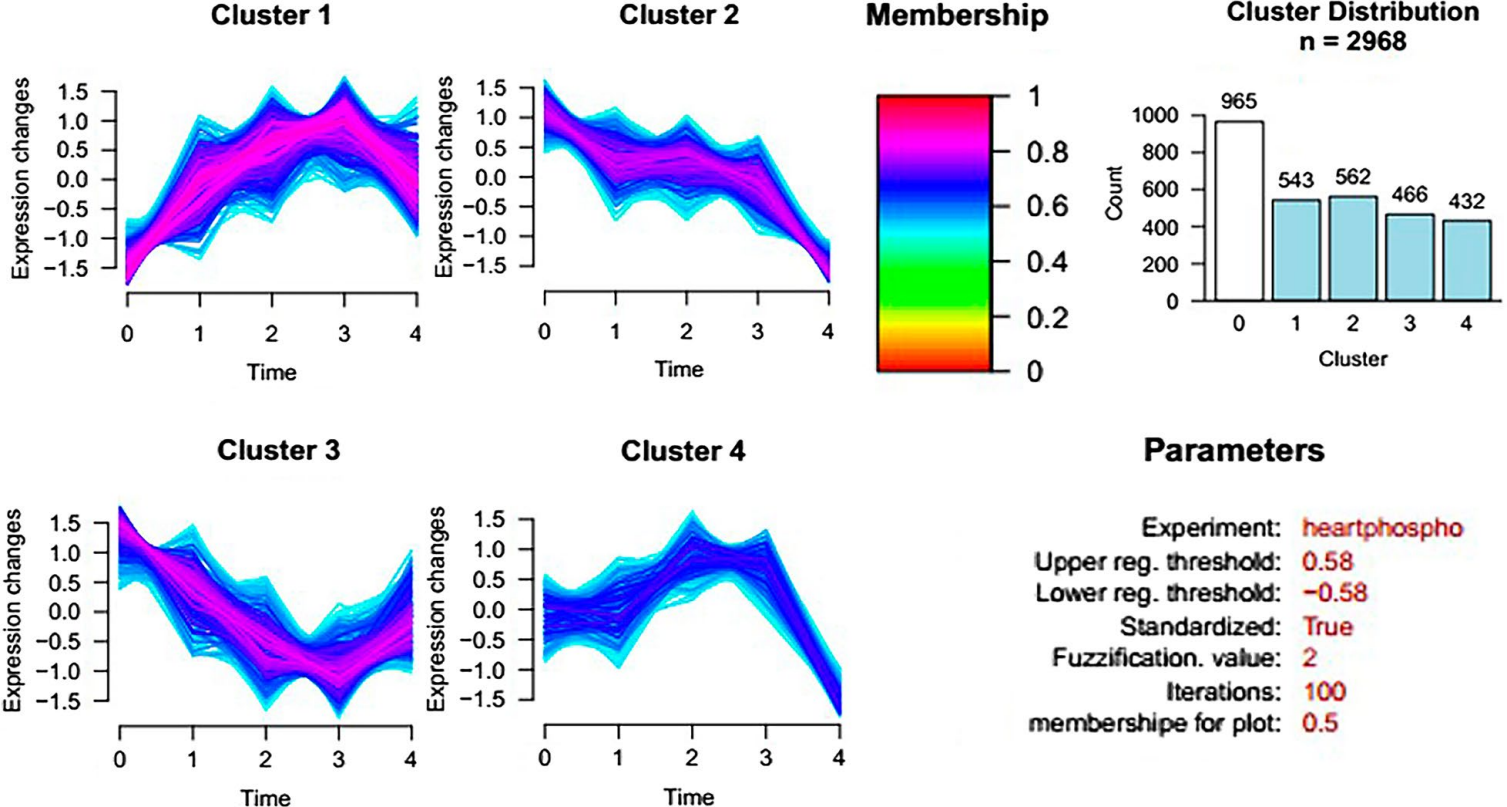
Cluster analysis of the phosphopeptide expression ratios of 5 days/0 days, 8 days/0 days, 11 days/0 days, and 19 days/0 days

Cluster analysis was performed on the phosphopeptide expression ratios of infected *B. microti* on 5 days/0 days, 8 days/0 days, 11 days/0 days, and 19 days/0 days, as shown in Fig. 9. Among them, 965 phosphopeptides in Cluster 0 were not differentially expressed, and the other 2003 were differentially expressed. There were four trends of differential expression of phosphopeptide modification. The numbers of phosphopeptides clustered into Cluster1–4 were 543, 562, 466, and 432, respectively. The degree of phosphorylated modification of peptides in Cluster 1 continuously increased until 11 days after *Babesia* infection. It showed that the phosphorylation level of these proteins continued to increase with the increasing infection rate of *B. microti* following initial infection. Cluster 2 has the most phosphopeptides, and the degree of modification continues to decrease following initial *B. microti* infection. The degree of phosphorylated modification of peptides in Cluster 3 decreased continuously when the infection rate increased, but began to increase at 11 days post-infection. The degree of phosphorylated modification of peptides in Cluster 4 did not change significantly over early stages of the infection, slowly upregulating from 5 days post-infection and downregulating from 11 days of infection.

### GO annotation of phosphorylated proteins in different clusters

**Fig. 10:**
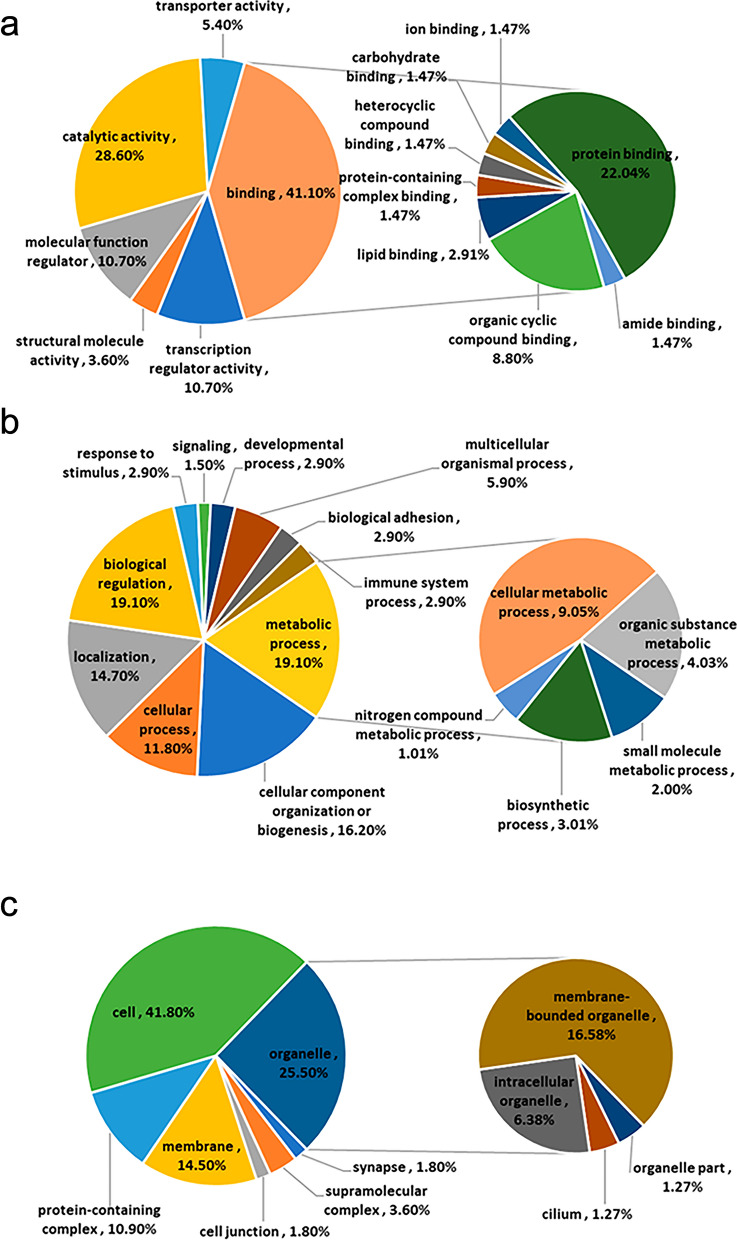
GO analysis of differential expressed phosphorylated proteins in Cluster 1. **a** Molecular function; **b** biological process; **c** cell component

GO function annotation was performed for phosphorylated proteins in different clusters, as shown in Fig. 10 and Additional file 4: Fig. S4, Additional file 4: Fig. S5, Additional file 4: Fig. S6. In each group, the differential proteins were mainly located in organelles, especially in membrane-bounded organelles. The functional analysis of various differential proteins showed that the proteins in Cluster 1 were mainly related to binding activity, particularly, the binding between proteins and organic cyclic compounds. These were mainly involved in material metabolism, biological regulation, localisation, and organisation and biosynthesis of cell components, whereas proteins in Cluster 2 mainly have catalytic activities, including oxidoreductase, transferase, and hydrolase. These were mainly involved in metabolic processes, including organic metabolism, biosynthesis, and small molecule metabolism. The proteins in Cluster 3 were also mainly related to binding activity, in which protein binding activity and travel cycle compound binding activity account for the majority of proteins; these were mainly involved in intracellular metabolism, biosynthesis and organic substrate metabolism. The protein function in Cluster 4 was similar to that in Cluster 1, mainly involved in metabolic processes, including organic substrate metabolism and biosynthesis with an emphasis on binding activity, in particular, binding between proteins and the binding of organic cyclic compounds.

### KEGG annotation of phosphorylation modification changed proteins in different clusters

**Fig. 11:**
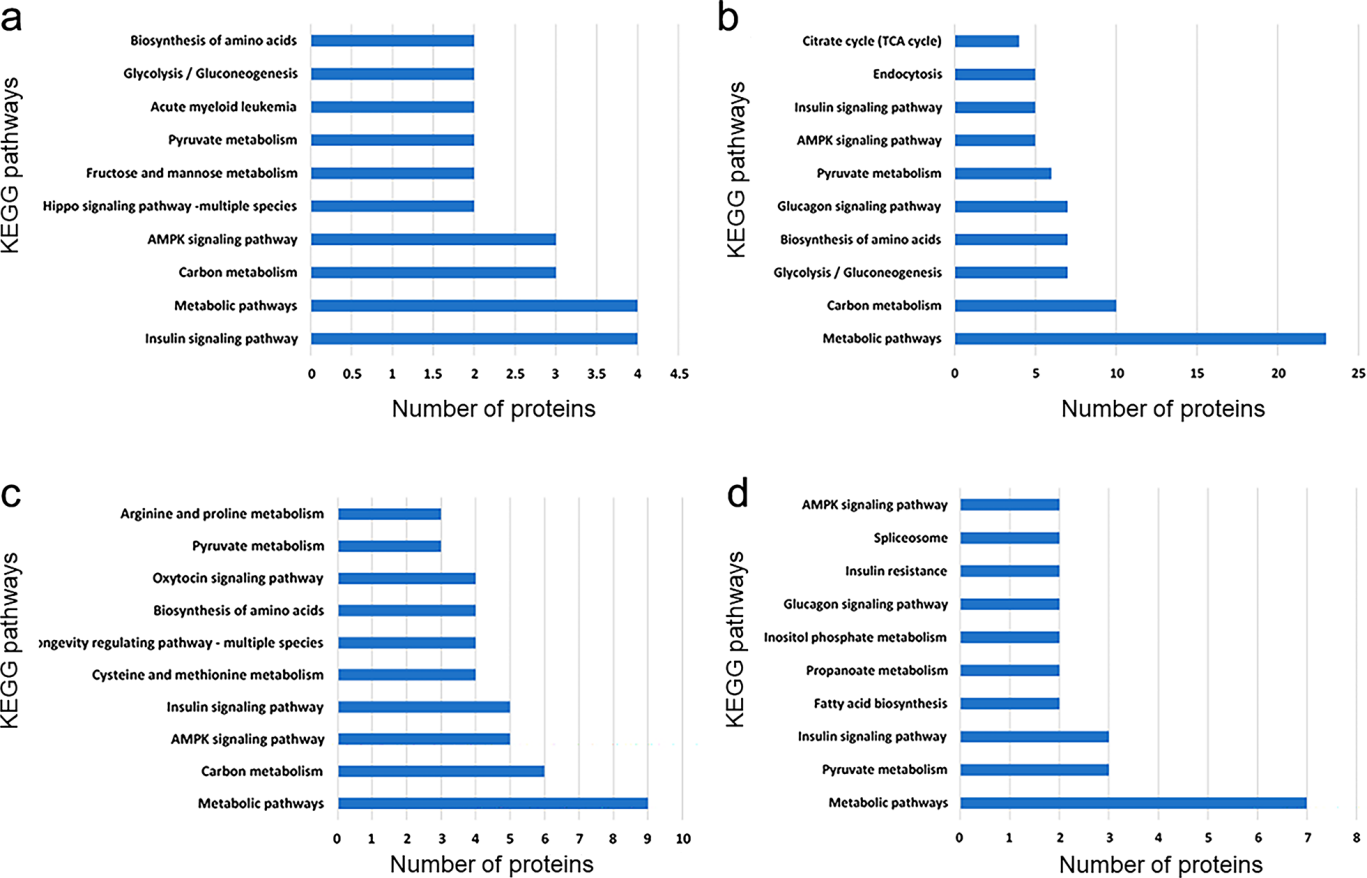
KEGG pathway analysis and quantitative statistics for differentially expressed phosphorylated proteins in each cluster. **a** KEGG enrichment results of proteins in Cluster 1; **b** KEGG enrichment results of proteins in Cluster 2; **c** KEGG enrichment results of proteins in Cluster 3; **d** KEGG enrichment results of proteins in Cluster 4

The differential proteins in Clusters 1–4 were enriched by KEGG pathway and the number of pathways were noted (as shown in Fig. 11). Four proteins in Cluster 1 participate in insulin signaling pathway and four proteins participate in metabolic pathway. Additionally, 23, 9, and 7 proteins in Cluster 2, Cluster 3, and Cluster 4 participate in the metabolic pathways, respectively.

*Babesia microti* infection most prominently affects energy metabolism-related signal pathways in mice. The differentially expressed proteins post-infection include Akap1, Prkaca, Prkab2, Acaca, Acacb, Aka1, Abcf1, Synj1, Braf, Vcl, and Fhod3, which involve insulin receptor signal, glucagon signal pathway, AMPK signal pathway, and Hippo signal pathway. With the participation of these signal pathways, the protein phosphorylation level related to many metabolic processes was also affected, such as amino acid synthesis, glycolysis/gluconeogenesis, pyruvate metabolism, fructose mannose metabolism, as well as cell division, cell migration, cell autophagy, and apoptosis-related proteins.

## Discussion

The heart is one of the most critical organs of the body powering blood circulation. It has been found that *Babesia* infection can lead to varying degrees of heart tissue damage, such as inflammation, microthrombus, myocardial infarction, subepicardial and subendocardial haemorrhage, and cardiac necrosis [31, 32]. It was shown that these cardiac changes have influence on disorders in circulation, and probably these are cause of death in infected animals [33]. In this study, to reveal the causes of cardiac damage by *B. microti* infection and the mechanisms of cardiac self-repair at the molecular level, the dynamic changes in protein expression and protein phosphorylation in the heart tissues of *B. microti* infected mice were systematically analysed using the DIA quantitative proteomics technique. The results showed that after *B. microti* infection the differentially expressed proteins in mice mainly include fibrinogen α (Fgα), fibrinogen β (Fgβ), Serpina1b, Serpina1c, cathepsin Z, cytochrome c oxidases (COXs), RPS11, and RPS20, while the proteins with phosphorylation changes mainly include 20-kDa light chain of myosin II (MLC20), Myosin light chain kinase (MLCK), mitogen-activated protein kinase 14 (MAPK14), and Akt1. These proteins were mainly involved in coagulation processes [34–37], cell apoptosis [38–41], oxidative phosphorylation [42], and ribosomes [43] (Table 1).

**Table 1:** Proteins related with heart damage and self-repair after *B. microti* infection in mice

Associated process	Protein name	Accession	5 d	8 d	11 d	19 d
Coagulation	Fgα	E9PV24	**↑**	**–**	**–**	**–**
	Fgβ	Q3TGR2	**↑**	**–**	**–**	**–**
	Serpina1b	P22599	**–**	**↓**	**↓**	**–**
	Serpina1c	A0A0R4J0X5	**–**	**↓**	**↓**	**↓**
Apoptosis	MLC20^℗^	D3YV37	**↓**	**↓**	**↓**	**↓**
	MLCK^℗^	B1B1A8	**–**	**–**	**↓**	**↓**
	Cathepsin Z	Q545I6	**–**	**–**	**↑**	**–**
	MAPK14^℗^	B2KF34	**–**	**↑**	**↑**	**↑**
	Akt1^℗^	D3YXX3	**–**	**↑**	**↑**	**–**
Oxidative phosphorylation	COX1	A0A286T4B5	**↓**	**–**	**↓**	**–**
	COX4i1	A2RSV8	**↓**	**–**	**↓**	**–**
	COX5a	P12787	**↓**	**–**	**↓**	**–**
	COX6b1	P56391	**↓**	**–**	**↓**	**–**
	COX6c	Q9CPQ1	**↓**	**–**	**↓**	**–**
Ribosome	RPS11	E9PYL9	**–**	**↑**	**–**	**–**
	RPS20	A0A140T8	**–**	**↑**	**↑**	**–**

**↑**, Upregulated; **↓**, downregulated; **–**, no significant change; ^℗^, phosphorylated

### Protein-related coagulation cascades

In mice, *B. microti* mainly infects RBCs and causes lysis of the cells. Upon infection, the body can activate the coagulation cascade and promote blood coagulation; this activation can lead to microthrombi in the blood vessels of the heart, myocardial infarction, and bleeding of the heart; such changes were observed in animals infected with various *Babesia* species such as *B. rossi*, *B. canis*, and *B. bigemina*. These observations may explain cardiac pathology observed in these previous studies [31–33]. This study revealed that after the mice had been infected with *B. microti*, proteins involved in complement formation, coagulation cascades, and platelet activation underwent changes in expression levels, indicating that local thrombosis in the heart may have occurred. The outcomes explained the pathogenesis of disseminated intravascular coagulation induced in human babesiosis [9].

Fibrinogen is a hexamer glycoprotein (GP) involved in the coagulation process and consists of three chains (α, β, and γ) [34, 35]. During blood coagulation, thrombin cleaves soluble fibrinogen into fibrin monomers; after polymerisation, the monomers assemble into fibrin polymers that interact with calcium ions and coagulation factor XIII to form stable fibrin clots and promote thrombosis [44]. Fibrinogen can also bind to the platelet GPIIb/IIIa receptors, connect adjacent platelets, act as a bridge to aggregate platelets, and promote blood clotting [45]. In addition, fibrinogen can promote the growth, proliferation, and contraction of vascular smooth muscle and endothelial cells [46]; increase blood viscosity and peripheral resistance; induce endothelial cell damage [47]; and promote the synthesis of collagen and DNA [48], the chemotactic migration of monocytes and macrophages to the intima [49, 50], and RBC adhesion and thrombosis [51, 52]. Abnormal fibrinogen expression can cause a variety of cardiovascular diseases. It was observed that the concentration of fibrinogen was increased in *Babesia*-infected animals [53]. Most patients with elevated fibrinogen levels are prone to myocardial infarction or sudden death, and the higher the fibrinogen level is, the greater the risk of developing disease [54–56]. Therefore, fibrinogen can serve as a marker for the clinical diagnosis of cardiovascular diseases [57]. In this study, the investigation of changes in cardiac proteins revealed that the expression levels of Fgα and Fgβ were increased significantly on day 5 after *B. microti* infection. The risk of local microthrombosis may occur in the heart of mice at this time.

Serine proteases, which are proteolytic enzymes with serine as their active centre, participate in many important activities in vivo, including coagulation. Common serine proteases involved in the coagulation process include thrombin, coagulation factor X, coagulation factor XI, coagulation factor Xa, and fibrinolysin. As a serine protease activity regulator, serpin can inhibit the activity of these enzymes and thereby inhibit coagulation [36, 37]. This study revealed that the expression levels of Serpina1b and Serpina1c in the hearts of mice were significantly reduced from day 8 to day 11 after *B. microti* infection, indicating that the inhibitory effect of serine protease inhibitors on the coagulation process was decreasing at this time. This decrease resulted in the intensification of blood coagulation in the heart microvessels, which can led to the formation of microthrombi in the heart. These changes were observed in animals infected with various *Babesia* species [31–33].

### Apoptosis-related proteins

**Fig. 12:**
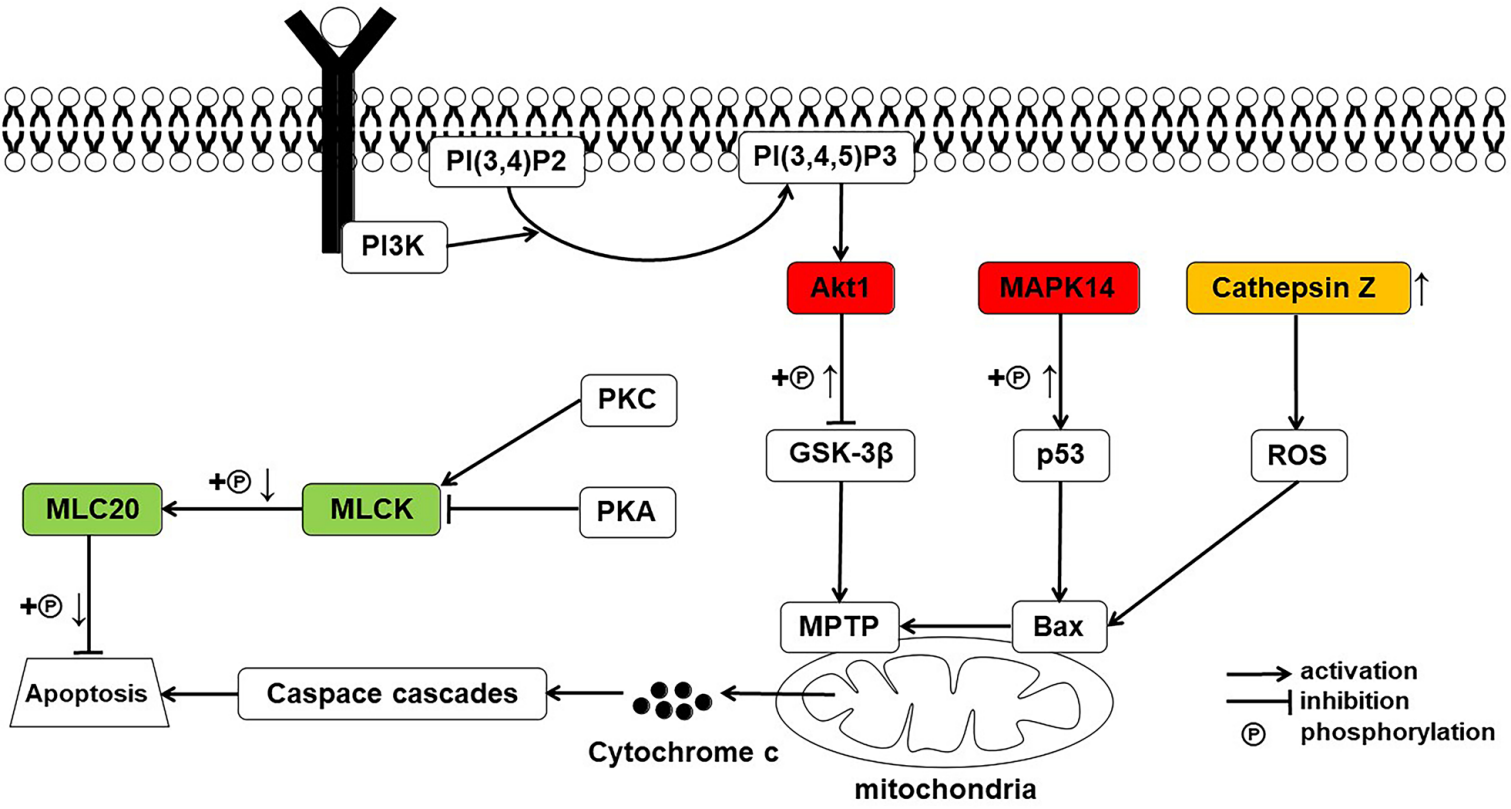
Simplified pathway diagram of apoptosis. “ + ℗ **↑**” indicates upregulated phosphorylation level of protein; “ + ℗ **↓**” indicates downregulated phosphorylation level of protein; “**↑**” indicates upregulated expression level of protein

Cell apoptosis is the most basic biological process by which multicellular organisms maintain their structural stability and functional balance of the internal environment and regulate growth and development; it is also an active and programmed physiological process for removing aged, aberrant, or deteriorated cells from the body. In addition, cell damage by pathogens or toxic substances can trigger apoptosis [58]. In this study, we found changes in expression and phosphorylation of proteins that participate in cell apoptosis (Fig. 12).

When cell apoptosis occurs, cell morphology undergoes a series of characteristic changes, with corresponding changes occurring in the cytoskeleton. The cytoskeleton, which includes microfilaments, microtubules, and intermediate filaments, is a component of eukaryotic cells important for maintaining life activities. Microfilaments are composed of actin, actin-binding protein, and myosin. Studies have found that prolonged dephosphorylation of the MLC20 can induce apoptosis [39]. MLCK is a calmodulin-dependent enzyme that catalyses the phosphorylation of MLC20, and the inhibition of this enzyme can induce cell apoptosis [39]. This study showed that the phosphorylation level of MLC20 protein in the heart continued to be downregulated after *B. microti* infection, which may explain the intensification of cardiac apoptosis. In addition, after *B. microti* infection, the phosphorylation level of MLCK protein was downregulated on day 11 and day 19; the function of this protein was likely inhibited at this time and thus unable to phosphorylate MLC20 effectively, inducing cell apoptosis. Apoptosis is closely related to many cardiovascular diseases. If cell apoptosis is excessive, it can induce heart failure and myocardial infarction [59]. In this study, after *B. microti* infection in mice, cardiomyocyte apoptosis was intensified, which might have caused myocardial infarction and heart failure in the host after infection.

The lysosome is a common organelle in animal cells and can decompose biomacromolecules, such as proteins, nucleic acids, and polysaccharides [60]. Lysosomes contain cathepsins [60], which play an important role in the regulation of apoptosis [61]. Cathepsins participate in apoptosis mainly via three molecular mechanisms. First, they participate in cell apoptosis through classical pathways, such as the death receptor pathway and mitochondrial pathway. Second, they participate through inflammatory mediators, some inflammatory cells, and other inflammatory responses. Third, they participate through the reverse blocking of the survival signalling pathway to activate the apoptotic signalling pathway [61]. Cathepsin Z is a member of the cysteine cathepsin protease family; the increase in the expression of cathepsin Z can increase reactive oxygen species (ROS) production, which results in mitochondrial dysfunction, Bax/Bak-mediated mitochondrial membrane permeabilisation, and release of cytochrome c and caspase cascades, followed by cell apoptosis [38]. The present study showed that the expression of cathepsin Z in mice began to increase significantly on day 11 after *B. microti* infection. This may cause rapid apoptosis of the heart, which occurred at this time, and myocardial infarction and heart failure were likely to eventually be induced in infected mice.

MAPK pathway transduces signals from the extracellular environment to the nucleus in response to a variety of different stimuli and participates in various intracellular signaling pathways that control complex cellular processes, including proliferation, development, differentiation, transformation, and apoptosis [62]. In mammalian cells, three MAPK pathways have been well characterised: Extracellular signal-regulated kinase (ERK), C-Jun N-terminal kinase (JNK), and p38 MAPK. The p38 MAPK pathway [63] is involved in the regulation of various diseases. For example, when the cell senses the hypoxia signal, it will initiate a series of signal transductions when the signal is transmitted to MAPK 14. This kinase is activated by phosphorylation. Phosphorylated MAPK14 can activate the tumor suppressor p53 [45], subsequently activating Bax to permeabilise mitochondria and trigger apoptosis [46]. In this study, the phosphorylation modification level of MAPK 14 in mice heart tissues was upregulated from day 8 to day 19 after infection with *B. microti*. We speculate that hemolytic anaemia and hypotension caused by *Babesia* infection lead to insufficient blood and oxygen supply to the myocardium [64], and the hypoxic environment initiates the p38 MAPK signaling pathway to accelerate cell apoptosis, resulting in myocardial contractile dysfunction and myocardial damage.

The serine/threonine kinase Akt plays a key role in various signal transduction pathways regulating the process of physiology and disease [65]. For instance, the phosphoinositide 3-kinase (PI3K)/Akt pathway enhances cell survival by inhibiting apoptosis, making it the most vital signaling pathway for survival. Also, the synthesis of phosphatidylinositol (3, 4, 5)-trisphosphate (PIP3), which accumulates at the membrane and triggers Akt by phosphorylation, is catalysed by the activation of PI3K [65, 66]. Activated Akt can also inactivate glycogen synthase kinase-3 beta (GSK-3β) by phosphorylation, reducing the opening of mitochondrial permeability transition pore (MPTP), resulting in the reduction of mitochondrial damage and subsequent inhibition of myocardial apoptosis [41]. From this study, we found that the phosphorylation level of Akt1 (Akt) in the heart was upregulated 8 and 11 days after the mice were infected with *B. microti*. We speculate that Akt1 is activated under hypoxic conditions caused by cardia ischaemia following *B. microti* infection, ultimately inhibiting myocardial apoptosis to protect the myocardium. In short, the upregulation of Akt1 phosphorylation level confirms the molecular mechanism theory of regulation that cardiomyocytes protect themselves by reducing apoptosis in response to hypoxia and diseases.

### Oxidative phosphorylation-related proteins

Cardiomyocytes need to constantly release the energy stored in fatty acids and glucose to obtain enough energy to maintain the heart's mechanical movement. However, the heart can store little energy, so it needs to constantly harvest and release energy to maintain its nonstop mechanical movement. The underlying pathway mainly converts free fatty acids and glucose, transported by the blood to mitochondria, into acetyl-CoA, which then enters the Krebs cycle to carry out oxidative phosphorylation in the mitochondrial respiratory chain to produce a large amount of adenosine triphosphate (ATP) [67].

COX is the terminal enzyme of the mitochondrial respiratory chain and is located on the inner mitochondrial membrane. Its main function is to transfer electrons to O_2_ to generate H_2_O, arrange protons into the mitochondrial intermembrane space, and finally convert adenosine diphosphate (ADP) to ATP through ATP synthase. The COX complex contains multiple metal cofactors and subunits and comprises a group of macromolecular proteins [42]. The number of COX subunits varies among different organisms; there are 13 subunits in mammals. The COX1, COX2, and COX3 subunits are encoded by mitochondrial DNA; they are highly evolutionarily conserved and form a catalytic reaction centre. The COX4, COX5a, COX5b, COX6a, COX6b, COX6c, COX7a, COX7b, COX7c, and COX8 subunits are encoded by nuclear genes, which guide the correct assembly of COX and maintain its structural stability [42]. Anaemia in babesiosis results from the parasite action and immune response. Changes such as spherocytosis and decreased mean corpuscular volume with anisocytosis indicate immune-mediated hemolytic anaemia. These changes were observed in animals with babesiosis [53, 68]. Haemolytic anaemia leads to insufficient blood and oxygen supply to the heart, and it can affect the oxidative phosphorylation of cardiomyocytes. Reduced oxidative phosphorylation can affect cardiomyocyte insufficiency and diastolic function and lead to irreversible damage of cardiomyocytes, and continuous ischaemia and hypoxia can cause myocardial infarction [69]. This study revealed that the expression levels of multiple COX subunits (COX1, COX4i1, COX5a, COX6b1, and COX6c) in the heart tissues of *B. microti*-infected mice all showed decreasing trends. Decreased contents of COX subunits could affect their activities, reduce the efficiency of oxidative phosphorylation, and eventually lead to an insufficient energy supply for cardiomyocytes. Therefore, *B. microti* infection may cause an insufficient oxygen supply in cardiac cardiomyocytes, which is very dangerous for patients.

### Ribosomal proteins

The ribosome is a site of intracellular protein synthesis. It is composed of a large subunit with a sedimentation coefficient of 60S and a small subunit with a sedimentation coefficient of 40S. Its major components are ribosomal RNA (rRNA) and RP; the large and small subunits were named RPL and RPS according to their origins [43]. In this study, RP large (RPL6, RPL7, RPL10, RPL11, RPL12, RPL13, and RPL35) and small subunits (RPS6, RPS7, RPS8, and RPS9) were detected after *B. microti* infection. On day 8 after infection, the expression levels of RPS11 and RPS20 were upregulated to varying degrees. The reason for this finding might be that after *B. microti* infection, the apoptosis of heart cells began to intensify, causing the body to synthesise many proteins to produce new cells.

## Conclusions

We investigated the changes in protein expression and phosphorylation modification levels in heart tissues using DIA quantitative proteomics trying to explain the molecular mechanisms of the injury and self-repair in the heart after *B. microti* infection. The results showed that the expression of coagulation cascade-related proteins (Fgα, Fgβ, Serpina1b, Serpina1c), apoptosis-related proteins (cathepsin Z), oxidative phosphorylation-related proteins (COX1, COX4i1, COX5a, COX6b1, and COX6c), and the phosphorylation modification levels of apoptosis-related proteins (MLC20, MLCK, MAPK14) are all changed, which are responsible for the damage of heart after *B. microti* infection. The changes in expression of ribosome proteins (RPS11, RPS20) and phosphorylation modification levels of apoptosis-related proteins (Akt1) revealed part of the molecular mechanisms of self-repair in the infected heart. This research offers a wealth of new targets for further exploration into the pathogenesis of heart disease induced by *Babesia* infection and is of great significance for novel drug development and new opportunities for targeted therapies.

## Supplementary Information

**Additional file 1: Figure S1.** GO analysis of differential expressed proteins in Cluster 2. (a) Molecular function; (b) biological process; (c) cell component.

**Additional file 2: Figure S2.** GO analysis of differential expressed proteins in Cluster 3.

**Additional file 3: Figure S3.** GO analysis of differential expressed proteins in Cluster 4.

**Additional file 4: Figure S4.** GO analysis of differential expressed phosphorylated proteins in Cluster 2.

**Additional file 5: Figure S5.** GO analysis of differential expressed phosphorylated proteins in Cluster 3.

**Additional file 6: Figure S6.** GO analysis of differential expressed phosphorylated proteins in Cluster 4. (a) Molecular function; (b) biological process; (c) cell component.

**Additional file 7: Table S1.** The raw data of global proteins identified at different stages.

**Additional file 8: Table S2.** Information of intersecting global proteins used for cluster analysis.

**Additional file 9: Table S3.** Proteins in each cluster.

**Additional file 10: Table S4.** The information of phosphopeptides identified at different stages.

**Additional file 11: Table S5.** Information of intersecting phosphopeptides identified at different stages.

## Data Availability

The mass spectrometry proteomics data have been deposited in the ProteomeXchange Consortium (http://proteomecentral.proteomexchange.org) via the iProX partner repository with the dataset identifier PXD029452.

## Abbreviations

RBC: Red blood cell
HCD: High energy collision dissociation
DIA: Data-independent acquisition
ACN: Acetonitrile
TFA: Trifluoroacetic acid
RP-HPLC: Reversed phase high-performance liquid chromatography
DDA: Data-dependent acquisition
AGC: Automatic gain control
PCA: Principal component analysis
GO: Gene Ontology
KEGG: Kyoto Encyclopedia of Genes and Genomes
CV: Coefficient of variation
Fgα: Fibrinogen α
Fgβ: Fibrinogen β
COX: Cytochrome c oxidase
MLC20: 20-KDa light chain of myosin II
MLCK: Myosin light chain kinase
MAPK14: Mitogen-activated protein kinase 14
RP: Ribosome protein
GP: Glycoprotein
ERK: Extracellular signal-regulated kinase
JNK: C-Jun N-terminal kinase
PI3K: Phosphoinositide 3-kinase
PIP3: Phosphatidylinositol (3, 4, 5)-trisphosphate
GSK-3β: Glycogen synthase kinase-3 beta
MPTP: Mitochondrial permeability transition pore
ATP: Adenosine triphosphate
ADP: Adenosine diphosphate
rRNA: Ribosomal RNA
